# The Effect of Water-Soluble Alpinia Galanga Extract on Sleep and the Activation of the GABAAergic/Serotonergic Pathway in Mice

**DOI:** 10.3390/ph17121649

**Published:** 2024-12-08

**Authors:** Kazim Sahin, Ahmet Kayhan Korkusuz, Emre Sahin, Cemal Orhan, Besir Er, Abhijeet Morde, Muralidhara Padigaru, Ertugrul Kilic

**Affiliations:** 1 Department of Animal Nutrition, Faculty of Veterinary Medicine, Firat University, 23119 Elazig, Türkiye; corhan@firat.edu.tr; 2 Department of Physiology, School of Medicine, Istanbul Medipol University, 34810 Istanbul, Türkiye; akayhankorkusuz@gmail.com; 3 Department of Animal Nutrition, Faculty of Veterinary Medicine, Bingol University, 12000 Bingol, Türkiye; esahin@bingol.edu.tr; 4 Department of Biology, Faculty of Science, Firat University, 23119 Elazig, Türkiye; ber@firat.edu.tr; 5 Research and Development, OmniActive Health Technologies, Mumbai 400013, India; a.morde@omniactives.com (A.M.); m.padigaru@omniactives.com (M.P.); 6 Department of Physiology, Faculty of Medicine, Istanbul Medeniyet University, 34700 Istanbul, Türkiye; kilic44@yahoo.com

**Keywords:** caffeine, GABAergic, sleep quality, neurotransmitters, Alpinia galanga

## Abstract

**Background/Objectives:** With increasing interest in plant-based compounds that can enhance sleep quality without the side effects of caffeine, Alpinia galanga (AG) has emerged as a promising herbal supplement for improving mental alertness. This study assessed the impact of water-soluble AG extract on sleep quality; the activity of GABAergic, glutamatergic, and serotonergic receptors; and concentrations of dopamine and serotonin in the brains of mice. **Methods:** The study employed two experimental models using BALB/c mice to examine the impact of pentobarbital-induced sleep and caffeine-induced insomnia. In the first model, a set of 20 mice was assigned to four groups to assess the effects of pentobarbital (42 mg/kg) or pentobarbital with AG extract on sleep induction, with observations made 45 min post-administration. In the second model, 20 mice were divided into four groups to evaluate the impact of caffeine (25 mg/kg) alone or caffeine with varying doses of AG extract (61.25 or 205.50 mg/kg administered orally) on brain activity along with additional analyses on receptor proteins and neurotransmitters. **Results:** A higher dose of AG extract (205.50 mg/kg) significantly increased total deep sleep duration compared to the caffeine group (*p* < 0.0001). Furthermore, this dose extended sleep latency and suppressed GABAergic and glutamatergic receptor activity compared to the lower AG dose (*p* < 0.05). Additionally, the 205.50 mg/kg dose elevated serotonin and dopamine levels compared to caffeine (*p* < 0.0001), suggesting improved sleep quality alongside enhanced wakefulness. **Conclusions:** Our data indicate that a higher dose of AG extract improved sleep latency and duration by regulating GABAergic and glutamatergic receptors through the GABAergic/serotonergic pathway in mice.

## 1. Introduction

Sleep is a physiological resting state that various factors, including disease, stress, and noise, can disrupt. Prolonged sleep deprivation can result in several health consequences, including immune system dysregulation, behavioral changes, and emotional and cognitive impairment [1]. In modern society, intense lifestyles, emotional imbalance, psychiatric disorders, and aging have collectively led to a significant prevalence of sleep deprivation and associated neurological disorders [2]. These conditions place a considerable burden on healthcare resources [3].

γ-Aminobutyric acid (GABA) is the principal inhibitory neurotransmitter in the central nervous system (CNS). Phasic inhibition of GABAergic signaling is initiated by the release of presynaptic GABA, which stimulates GABA_A_ and GABA_B_ receptors in the postsynaptic and presynaptic membranes [4]. Research has firmly established that the activation of GABA receptors contributes to sleep promotion [5]. GABAergic neurons facilitate non-rapid eye movement (NREM) sleep by interacting with the sublaterodorsal nucleus and lateral hypothalamus [6]. The inhibition of GABAergic signaling is a significant factor in the adverse effects of sleep latency and prolonged sleep duration [7]. Glutamate is implicated in initiating and maintaining sleep/wake cycles. It modulates muscle tone during wakefulness while mediating rapid eye movement (REM) sleep [8]. REM sleep deprivation could impede the glutamatergic signaling system by decreasing the N-methyl-d-aspartate receptors (NMDARs) and AMPA receptor subunit 1 (GluR1) [9].

Dietary factors are closely related to sleep quality and latency [10]. Caffeine-containing drinks such as coffee and tea have been found to delay sleep onset and decrease total sleep time [11]. However, prolonged caffeine intake has an abuse potential and may induce psychological and physical dependence [12]. Caffeine can interact with GABAergic pathways and receptors [13]. It transiently reduces inhibitory postsynaptic currents in GABAergic pathways associated with CA1 pyramidal cells in the hippocampus [14]. Because caffeine blocks adenosine A receptors in the brain, it can suppress the GABAergic receptors, particularly GABA_A_ [13]. Thus, there has been an increasing desire for caffeine-free plant-derived compounds that rapidly enhance sleep quality and alertness without the adverse consequences of caffeine consumption.

The Zingiberaceae family member *Alpinia galanga* (AG) has traditionally been used to treat several diseases. AG extracts can exert neuroprotective, gastroprotective, anti-allergic, anti-psoriatic, anti-microbial, anticancer, antioxidant, and anti-inflammatory effects, mainly owing to their phytoconstituents, including phenyl prostanoids, flavonoids, and terpenoids [15]. Earlier reports have demonstrated that AG extract effectively stimulates the CNS [16] and enhances focus and attention [17] by exerting a beneficial impact on mental alertness compared to caffeine [18]. However, the effect of EnXtra, a proprietary extract of AG (E-AG-01), on sleep and its mechanisms has yet to be explored. This study investigates the effects of water-soluble Alpinia galanga extract (EnXtra) on sleep patterns in a pentobarbital-induced sleep model in mice. Specifically, it seeks to assess whether EnXtra enhances sleep quality and modulates sleep architecture via the GABAergic system, offering potential as a plant-derived alternative for improving sleep without the adverse effects associated with caffeine.

## 2. Results

### 2.1. Sleep Duration and Latency

**Figure 1:**
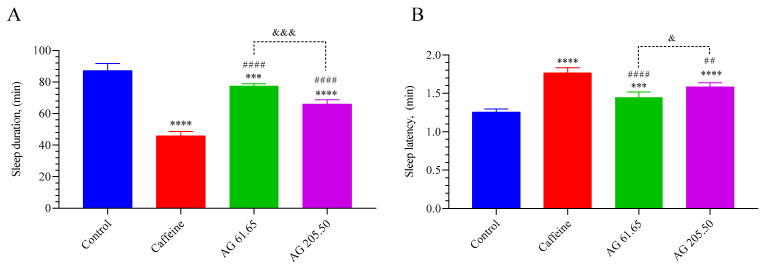
The effects of Alpinia galanga extract (EnXtra) on sleep duration (**A**) and sleep latency (**B**) in mice treated with pentobarbital. Each bar represents the mean and standard deviation. Different symbols are utilized to denote the statistical differences observed between groups (*** *p* < 0.001, **** *p* < 0.0001 as compared with the control group; ^##^ *p* < 0.01, ^####^ *p* < 0.0001 as compared with the caffeine group; ^&^ *p* < 0.05, ^&&&^ *p* < 0.001 as compared with the AG 61.65 group) using ANOVA and Tukey’s post hoc test. Control: Mice were treated with a hypnotic dose (45 mg/kg, i.p.) of pentobarbital; Caffein: 25 mg/kg caffeine was administered via oral gavage; AG: 61.65 and 205.50 mg/kg Alpinia galanga extract were administered orally to the mice 45 min before pentobarbital.

As presented in Figure 1, the control group demonstrated significantly longer sleep duration compared to all other groups (*p* < 0.001 for all). Additionally, a higher dose of AG (205.50 mg/kg) significantly reduced sleep duration compared to the lower dose (61.65 mg/kg) (*p* < 0.001). In contrast, as anticipated, the control group had a shorter sleep latency compared to the other groups (*p* < 0.001 for all). The caffeine group displayed the most prolonged sleep latency, which was significantly higher than both the AG 61.65 (*p* < 0.001) and AG 205.50 groups (*p* < 0.01). Furthermore, the high dose of AG was associated with an increase in sleep latency compared to the low dose (*p* < 0.05).

### 2.2. Wakefulness and NREMS

**Figure 2:**
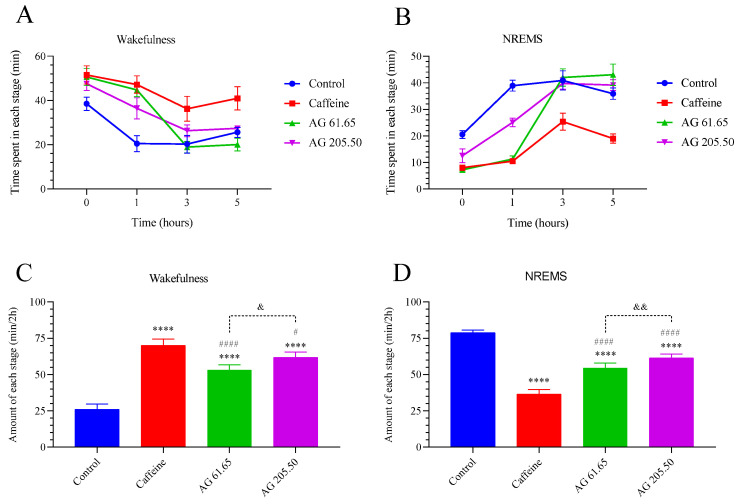
The effects of Alpinia galanga extract (EnXtra) on the time spent in each stage of wakefulness (**A**), time spent in each stage of non-rapid eye movement sleep (NREMS) (**B**), amount of each stage in wakefulness (**C**), and amount of each stage in NREMS (**D**). Each bar represents the mean and standard deviation. Different symbols are utilized to denote the statistical differences observed between groups (**** *p* < 0.0001 as compared with the control group; ^#^ *p* < 0.05, ^####^ *p* < 0.0001 as compared with the caffeine group; ^&^ *p* < 0.05, ^&&^ *p* < 0.01 as compared with the AG 61.65 group) ANOVA and Tukey’s post-hoc test. Control: Untreated mice; Caffein: Caffeine 25 mg/kg was administered via oral gavage; AG: Alpinia Galanga extract 61.65 and 205.50 mg/kg were administered orally.

At two-hour intervals (initial, 1st, 3rd, and 5th hours), the time spent in each stage of wakefulness and the time spent in each stage of NREMS were measured to determine the amount of each stage in wakefulness and the amount of each stage in NREMS (Figure 2). Caffeine treatment, acting as a stimulant, significantly increased the amount of wakefulness at each stage compared to the other groups (*p* < 0.001, for all). Additionally, the AG 205.50 group showed a higher wakefulness duration compared to the AG 61.65 group (*p* < 0.05). In contrast, caffeine administration notably decreased the total duration of NREMS. While both AG doses were more effective than the control group in preserving NREMS, neither dose had as profound an impact on NREMS duration as caffeine (*p* < 0.0001). Additionally, the AG 205.50 group showed a stronger effect on increasing NREMS duration compared to the AG 61.65 group (*p* < 0.01).

### 2.3. Brain Serotonin and Dopamine

**Figure 3:**
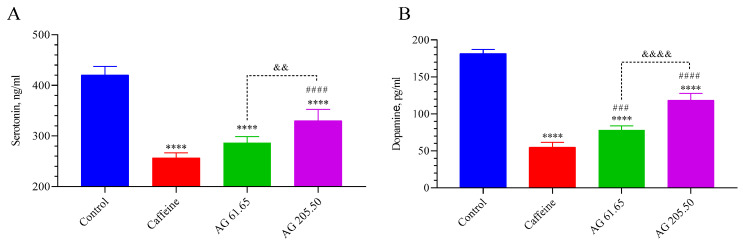
The effects of Alpinia galanga extract (EnXtra) on brain levels in serotonin (**A**) and dopamine (**B**). Each bar represents the mean and standard deviation. Different symbols are utilized to denote the statistical differences observed between groups (**** *p* < 0.0001 as compared with the control group; ^###^ *p* < 0.001, ^####^ *p* < 0.0001 as compared with the caffeine group; ^&&^ *p* < 0.01, ^&&&&^ *p* < 0.0001 as compared with the AG 61.65 group) using ANOVA and Tukey’s post hoc test. Control: Untreated mice; Caffein: 25 mg/kg caffeine was administered via oral gavage; AG: 61.65 and 205.50 mg/kg Alpinia galanga extract were administered orally.

As seen in Figure 3, upon administration of caffeine and AG (for low and high doses), the brain levels of serotonin and dopamine significantly decreased compared to the Control group (*p* < 0.0001, for all). The AG 61.65 and the caffeine groups had similar brain serotonin levels (*p* > 0.05). However, the AG 205.50 group had significantly greater serotonin levels than both the Caffeine (*p* < 0.0001) and the AG 61.65 group (*p* < 0.01). Brain dopamine levels were remarkably higher in the AG 61.65 and AG 205.50 groups compared to the caffeine group (*p* < 0.001 for all). The AG 205.50 group also showed significantly higher dopamine levels than the Caffeine and AG 61.65 groups (*p* < 0.0001).

### 2.4. GABAergic Receptors

**Figure 4:**
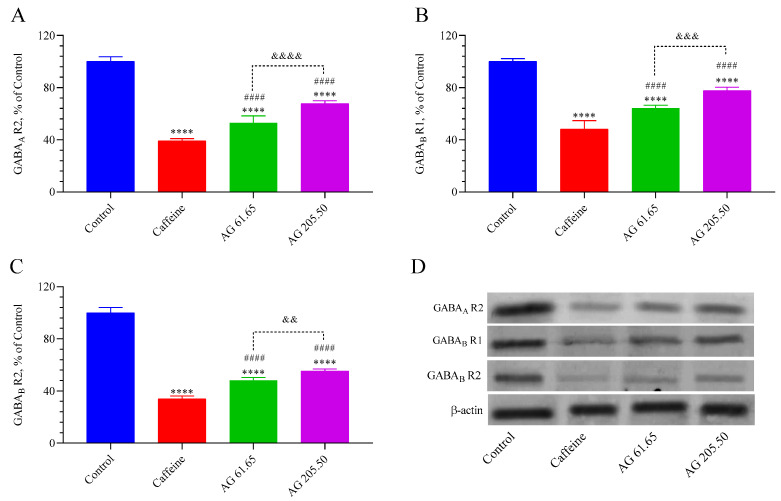
The effects of Alpinia galanga extract (EnXtra) on brain protein levels in GABAAR2 (**A**), GABABR1 (**B**), and GABABR2 (**C**). Also, the intensity of the Western blot bands (**D**) was quantified through a densitometric analysis, with β-actin included to confirm equal protein loading. The data are expressed as a ratio relative to the control value, which is assigned a baseline of 100%. Error bars represent the standard error of the mean. Different symbols are utilized to denote the statistical differences observed between groups (**** *p* < 0.0001 as compared with the control group; ^####^ *p* < 0.0001 as compared with the caffeine group; ^&&^ *p* < 0.01, ^&&&^ *p* < 0.001, ^&&&&^ *p* < 0.0001 as compared with the AG 61.65 group) using ANOVA and Tukey’s post hoc test. Control: Untreated mice; Caffein: was caffeine 25 mg/kg administered via oral gavage; AG: 61.65 and 205.50 mg/kg Alpinia galanga extract were administered orally.

The levels of GABA_A_R2, GABA_B_R1, and GABA_B_R2 receptors are presented in Figure 4. The findings indicate that caffeine and AG (for low and high doses) noticeably diminished the levels of these inhibitory receptors in the brain compared to the Control group (*p* < 0.0001). The AG 61.65 and AG 205.50 group demonstrated a remarkably superior enhancement in GABA_A_R2, GABA_B_R1, and GABA_B_R2 levels than the caffeine group (*p* < 0.0001), and the AG 205.50 group demonstrated even higher GABA_A_R2, GABA_B_R1, and GABA_B_R2 levels than the AG 61.65 group (*p* < 0.01, for all).

### 2.5. Glutamatergic and Serotonergic Receptors

**Figure 5:**
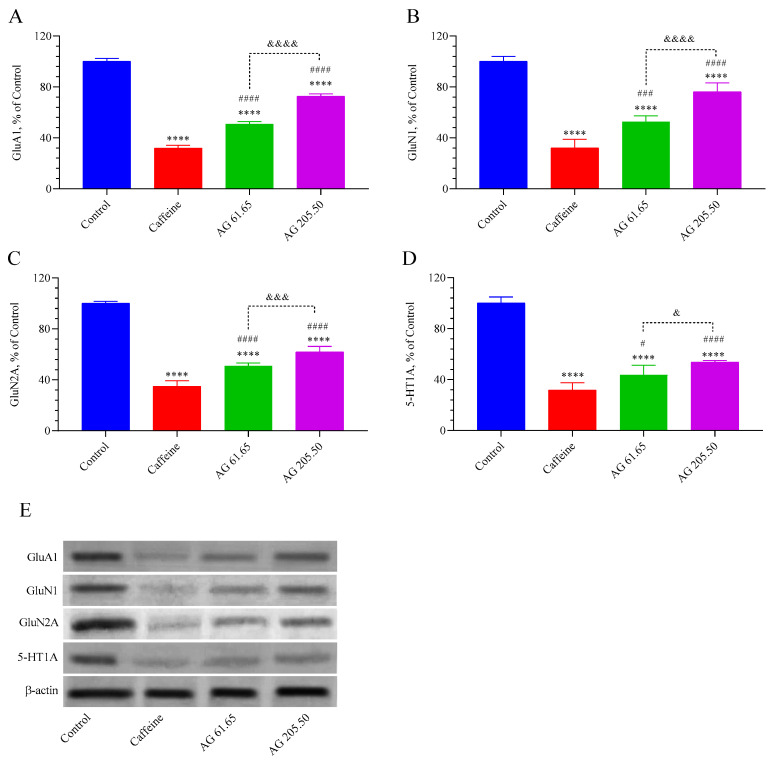
The effects of Alpinia galanga extract (EnXtra) on brain protein levels in GluA1 (**A**), GluN1 (**B**), GLuN2A (**C**), and 5-HT1A (**D**). Also, the intensity of the Western blot bands (**E**) was quantified through a densitometric analysis, with β-actin included to confirm equal protein loading. The data are expressed as a ratio relative to the control value, which is assigned a baseline of 100%. Error bars represent the standard error of the mean. Different symbols are utilized to denote the statistical differences observed between groups (**** *p* < 0.0001 as compared with the control group; # *p* < 0.05, ^###^ *p* < 0.001, ^####^ *p* < 0.0001 as compared with the caffeine group; ^&^ *p* < 0.05, ^&&&^ *p* < 0.001, ^&&&&^ *p* < 0.0001 as compared with the AG 61.65 group) using ANOVA and Tukey’s post hoc test. Control: Untreated mice; Caffein: 25 mg/kg caffeine was administered via oral gavage; AG: 61.65 and 205.50 mg/kg Alpinia galanga extract were administered orally.

The levels of GluA1, GluN1, GluN2A, and 5-HT1A receptors are given in Figure 5. We observed that caffeine and AG treatment decreased the levels of these receptors in the brain compared to the control group (*p* < 0.0001). Remarkably, the AG 61.65 and AG 205.50 groups exhibited higher levels of brain GluA1 (*p* < 0.0001 for all), GluN1 (*p* < 0.001, for all), and 5-HT1A (*p* < 0.05, for all) compared to the caffeine group. The AG 205.50 group showed higher GluA1(*p* < 0.0001), GluN1 (*p* <0.001), GluN2A (*p* < 0.0001), and 5-HT1A (*p* <0.05) levels than the AG 61.65 group.

### 2.6. Neurotrophic Factors

**Figure 6:**
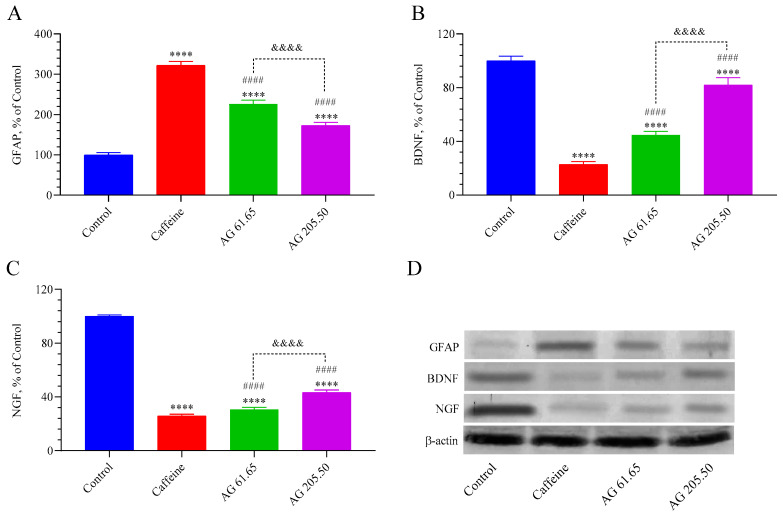
The effects of Alpinia galanga extract (EnXtra) on brain protein levels in GFAP (**A**), BDNF (**B**), and NGF (**C**). Also, the intensity of the Western blot bands (**D**) was quantified through a densitometric analysis, with β-actin included to confirm equal protein loading. The data are expressed as a ratio relative to the control value, which is assigned a baseline of 100%. Error bars represent the standard error of the mean. Different symbols are utilized to denote the statistical differences observed between groups (**** *p* < 0.0001 as compared with the control group; ^####^ *p* < 0.0001 as compared with the caffeine group; ^&&&&^ *p* < 0.0001 as compared with the AG 61.65 group) ANOVA and Tukey’s post-hoc test. Control: Untreated mice; Caffein: 25 mg/kg caffeine was administered via oral gavage; AG: 61.65 and 205.50 mg/kg Alpinia galanga extract were administered orally.

The GFAP, BDNF, and NGF levels in the brain are shown in Figure 6. Notably, all of the treated groups had higher levels of the GFAB protein than the control group (*p* < 0.0001), but all of the treated groups had lower levels of the NGF and BDNF proteins than the control group (*p* < 0.0001). The GFAB levels in the AG 61.65 and AG 205.50 groups were significantly lower than those in the caffeine group (*p* < 0.0001). Compared to the AG 61.65 group, the AG 205.50 group exhibited reduced GFAB levels (*p* < 0.0001). The AG 61.65 and AG 205.50 groups exhibited notably elevated levels of BDNF and NGF compared to the caffeine group (*p* < 0.0001). Furthermore, the AG 205.50 group exhibited elevated levels of BDNF and NGF compared to the AG 61.65 group (*p* < 0.0001).

## 3. Discussion

The current study evaluated the effects of EnXtra on sleep patterns, neurotransmitter levels, and receptor activity in mice, comparing them with caffeine and a control group. The findings demonstrate that both doses of AG extract significantly impacted sleep duration, latency, wakefulness, and NREMS as well as brain neurotransmitter levels, receptor activity, and neurotrophic factors. Based on previous studies showing that AG extract improves brain function and acts as a neuroprotective agent [18,19,20,21], we studied the effects of EnXtra at different doses on sleep quality and certain markers related to brain functions, such as GABAergic, glutamatergic, and neurotrophic factors, comparing them to the effects of caffeine in pentobarbital-induced sleep in mice.

AG has demonstrated anxiolytic effects in preclinical studies, potentially attributed to its ability to modulate neurotransmitter systems and reduce oxidative stress. For instance, bioactive compounds like galangin and 1′-acetoxycavicol acetate have diminished anxiety-like behaviors in animal models [22]. These effects may be mediated by the GABAergic system, which is integral to anxiety regulation. Furthermore, the anti-inflammatory properties of AG may contribute to its potential in managing amnesia, supported by increasing evidence that links inflammation to cognitive health [19].

The negative impact of caffeine on mice’s sleep duration and latency was described as decreasing in NREMS and increasing in wakefulness. Similarly, the administration of low or high doses of AG resulted in a reduction in the duration of sleep and the amount of NREMS. Nevertheless, administering 205 mg/kg AG extract showed greater efficacy in extending sleep latency and wakefulness and a higher amount of NREMS than caffeine. The current findings are strongly supported by a systematic review by Gardiner, et al. [23], which provides evidence that caffeine consumption significantly affects sleep onset, sleep efficiency, the maintenance of subsequent sleep, and overall subjective sleep quality. Previous studies have shown that the water-based extract of AG enhances wakefulness [17] and positively impacts several dimensions of cognitive function, such as mental vigilance, reaction time, and accurate responses, for up to five hours after administration [24]. It has been reported that various classes of compounds, including phenolic compounds and terpenes, were present in the phytochemical analysis of different parts of AG [15]. Studies consistently demonstrate that polyphenols enhance NREMS to a degree comparable to lower-dose benzodiazepines [25]. Also, some terpenoid compounds, such as α-pinen, can increase the intensity and duration of NREMS [26]. Hence, in the current study, the enhancement of NREMS by AG is likely attributed to its phytochemical composition. The findings also revealed that AG extract improved sleep quality in a dose-dependent manner. This extract may offer an alternative to caffeine, which promotes wakefulness by suppressing NREMS.

Concordantly with our results, Dasdelen, et al. [27] reported that caffeine administration decreased the brain sedative neurotransmitters dopamine and serotonin in mice. Serotonin serves as the precursor of melatonin, suggesting a potential function in initiating sleep-stimulating inhibitory neurons [28]. Serotonin is involved in both the facilitation and inhibition of sleep and the promotion of wakefulness. Its intricate impact on the sleep–wake cycle is linked to its influence on various brain regions involved in regulating sleep and wakefulness. Therefore, the serotonergic system in the brain can mediate hypnotic-sedative activity [29]. Although previous studies showed that most phytochemicals improve serotonin and dopamine levels [30], we observed that AG extract lowered brain serotonin and dopamine levels in mice with pentobarbital-induced sleep. These results show that AG can potentially increase sleep quality, even if it prolongs wakefulness, and may diminish the side effects of pentobarbital or other hypnotic drugs by modulating serotonergic and dopaminergic functions.

The regulation of sleep-inducing circuits by GABA receptors is well recognized, and their targeting for treating sleep-related disorders is well developed [31]. Pentobarbital exerts its pharmacological effects by stimulating GABA_A_ receptors, prolonging GABAergic inhibitory postsynaptic currents, and increasing the probability of channel opening in GABA_A_ receptors [32]. Herein, we found that caffeine and AG extract may provoke sleeplessness by blocking GABA_A_ and GABA_B_ receptors in the brain in mice. Although no research has been conducted on AG’s effect on GABA receptors, its phytochemical composition may be responsible for this effect. Preclinical and clinical studies demonstrated that phytochemicals differentially affect GABA receptor expression [33]. In a study by Wang, et al. [34], it was found that ginger, which belongs to the same family as AG, when administered, could inhibit the expression of the GABA receptor in hippocampal tissue, thus helping in the prevention of ethanol-induced cognitive impairment. In contrast, Rhizoma Curcumae, which contains curcumol, curcumin, and curdione, was found to enhance GABAergic inhibition by increasing GABA_A_ receptor-mediated tonic currents in hippocampal tissue, specifically through curcumol [35]. For the first time, we reported that the AG extract reduces GABA receptor activities less than caffeine, which probably enhances sleep quality more than caffeine.

Caffeine enhances the release of neurotransmitters and inhibits the activity of AMPA-type glutamate-activated channels in the brain [36]. Consistent with previous research [27], our findings indicate that caffeine-induced sleep deprivation significantly decreases GluA1, GluN1, and GluN2a levels. The observed reduction in glutamate receptor levels suggests a potential mechanism through which the AG extract may contribute to improved sleep quality while decreasing sleep duration within pentobarbital-induced sleep in a dose-dependent manner. The NMDA receptor exerts stimulatory regulation of neuronal GABA release [37]. Previously, Ostrowski, et al. [38] reported that GABA and glutamate synaptic signaling can be depressed by serotonergic receptor 5-HT_1A_. Conversely, our result indicated that brain 5-HT_1A_ receptor levels were suppressed with GABA and glutamate receptors in pentobarbital-induced sleep mice. Parallelly to our findings, Sahin, et al. [39] showed that the activity of the serotonergic synaptic signaling pathway influences the expression of 5-HT and GABA in the mice hippocampus.

The balance between excitatory and inhibitory functions is crucial for properly functioning the brain’s physiological processes, including regulating the sleep–wake cycle and its intensity and duration. Maintaining equilibrium between the GABAergic and glutamatergic systems is vital for adequate sleep [40]. This study shows that AG extracts stimulate both GABA and glutamate receptors, resulting in increased sleep latency and improved sleep quality, as evidenced by a significant extension of NREMS duration. However, the lack of extensive preclinical or clinical research complicates the mechanistic explanations. (1) Unlike caffeine, higher doses of AG may regulate the synaptic accumulation of glutamate and GABA by slightly modulating glutamatergic and GABAergic receptors. The chemical diversity of herbal compounds allows them to interact with multiple binding sites on GABAA receptors, potentially leading to positive or negative modulation [41]. It is plausible that AG’s chemical components interact with distinct binding sites on these receptors, thereby influencing GABA release [41]. (2) Serotonin receptor subtypes also play a role in modulating GABA and glutamate release. The activation of the 5HT1A receptor may inhibit GABA and glutamate release, decreasing neuronal inhibition and potentially increasing neural excitability. Conversely, 5HT3 receptor activation enhances GABA and glutamate release in specific brain regions, contributing to greater inhibitory signaling [42]. Given the chemical constituents of AG, it is likely that both 5HT1A and 5HT3 receptors are involved, differentially regulating the GABAergic and glutamatergic systems to maintain excitatory neurotransmission balance. (3) NREM and REM sleep duration are closely linked to GABAergic stimulation [43]. GABAergic activation predominantly supports NREM sleep, with GABAB receptors promoting NREM sleep through prolonged inhibitory effects. These receptors may enhance slow-wave sleep (SWS), characteristic of deep sleep, by reducing communication between the brain’s thalamus and cortex [44]. AG extract appears to upregulate GABAB receptors dose-dependently, potentially promoting NREM sleep in mice. (4) Serotonin is critical in initiating and maintaining NREM sleep while suppressing REM sleep. Its activity declines during the deeper stages of the NREM cycle [45]. Serotonergic signaling helps regulate excessive NREM sleep rebound, ensuring homeostasis following sleep deprivation [46]. Dopamine signaling increases significantly during the transition from NREM sleep to wakefulness [47]. Higher doses of AG seem to enhance serotonin and dopamine levels in the brain, contributing to increased NREM sleep and reduced total sleep duration. The observed rise in the proportion of NREM sleep relative to total sleep time suggests that elevated doses of AG may be an effective strategy for improving sleep quality.

The activity of the serotonergic and dopaminergic pathways is thought to be influenced by BDNF, one of the most significant neurotrophins in the brain. Lower serum BDNF level is linked to sleep deprivation [48]. Similarly, serum NGF levels were substantially lower in adolescents with poor sleep quality [49]. In contrast, serum GFAP levels may increase in individuals experiencing sleep disorders, such as insomnia [50]. The current study indicates that caffeine administration led to a decrease in brain BDNF and NGF levels while simultaneously increasing GFAP levels. Upon analysis, it was observed that the brain’s GABAergic receptors, serotonin levels, and dopamine levels were interrelated, providing evidence for AG extract’s regulatory impact on these neurochemical factors. Galangin can mitigate the doxorubicin-provoked suppression of hippocampal BDNF while upregulating GFAB, thus preventing cognitive decline in rats [51]. In addition, chronic curcumin supplementation, the main constituent of Zingiberaceae plants, leads to a dose-dependent increase in hippocampal BDNF in depressed rats [52]. On the other hand, Ruan, et al. [53] demonstrated that curcumin supplementation inhibited relative hippocampal BDNF expression in mice with sleep deprivation. The studies above posit that flavonoids may have differential effects on regulating BDNF and related neuronal factors. Thus, our findings indicate that the AG extract may exert varying modulatory effects on BDNF-related GABA transmission in pentobarbital-induced sleep in mice.

While this study provides valuable insights into the effects of AG extract on sleep patterns, neurotransmitter levels, and receptor activities, it is important to note that these analyses were conducted on whole-brain samples. This approach does not allow for the differentiation of region-specific effects, which are particularly relevant given the opposing roles of neurotransmitter receptors, such as 5-HT1A, in distinct brain regions (e.g., presynaptic receptors in the midbrain versus postsynaptic receptors in the cortex or hippocampus). Future studies should focus on region-specific investigations to better elucidate the molecular mechanisms underlying AG’s effects. Techniques such as region-specific RNA/protein analyses and in vivo imaging may help clarify these pathways and strengthen the findings of this study.

In conclusion, the present results show that AG extract doses of 61.65 mg/kg and 205.50 mg/kg to mice effectively prolonged wakefulness. Although AG extract decreased sleep duration, it improved sleep quality and related neurochemical changes. The modulation of these neurochemical changes was primarily attributed to GABAergic and serotonergic pathways. Based on the current findings, it is suggested that the AG extract may positively impact mental alertness and productivity without causing the crash or sleep disturbances often associated with caffeine. However, additional research is necessary to elucidate the mechanisms driving these effects and to investigate their potential clinical significance.

## 4. Materials and Methods

### 4.1. Animals

A total of 40 male BALB/c mice, aged 8 weeks and weighing approximately 20 ± 3 g, were housed in a controlled environment with a 12:12 h light–dark cycle at a temperature of 22 °C. The mice were provided with chow and water ad libitum. All experiments were conducted in accordance with the National Institutes of Health’s Guidelines for the Care and Use of Laboratory Animals. Additionally, ethical approval was obtained from the Local Ethics Committee for Animal Experiments at Firat University and Medipol University, ensuring compliance with all relevant standards and regulations. This study was designed and reported in accordance with the Animal Research: Reporting of In Vivo Experiments (ARRIVE) guidelines. The sample size was determined by power analysis using G*Power. To achieve a statistical power of (1 − β), the analysis indicated that a total sample size of 40 (N) was required, with an effect size of 0.60 (f) and a type I error rate (α) of 0.05.

### 4.2. Experimental Procedures and Sampling

The pentobarbital-induced sleep test was performed in adherence to the procedure established by Cho, et al. [54] and was conducted between 13:00 and 17:00, with the mice subjected to a 24 h fasting period beforehand. Mice were allocated randomly into four groups, each comprising five mice: (i) the control group, treated with a hypnotic dose of pentobarbital (45 mg/kg intraperitoneally); (ii) the caffeine group, treated with pentobarbital and given 25 mg/kg caffeine via oral gavage; (iii) AG 61.25 group, treated with pentobarbital and given 61.65 mg/kg of EnXtra via orally; and (iv) AG 205.50 group, treated with pentobarbital and given 205.50 mg/kg of EnXtra via orally. Pentobarbital was diluted in 0.9% physiological saline. EnXtra was suspended in 0.01% DMSO.

After being given pentobarbital, the mice were put in separate cages, and their sleep duration and latency were tracked. Caffeine and EnXtra were given to the mice orally 45 min prior to the administration of pentobarbital. The observers were blinded to the other treatment groups to ensure objectivity and eliminate any potential bias. Sleep duration was determined by measuring the interval between the loss and recovery of the righting reflex, and sleep latency was measured as the amount of time between the pentobarbital injection and the start of sleep.

EnXtra, AG extract (with a concentration of 94–97%, comprising 4.87% polyphenols, 4.75% flavonoids, 39.09% glycosides, and 4.85% tannins) was derived from taxonomically authenticated AG rhizomes through the process of DNA barcoding. The extract is water-soluble, free of methyl eugenol, and standardized for polyphenols, polysaccharides, and pyrocatechol-type tannins. EnXtra was supplied by OmniActive Health Technologies (Mumbai, India).

The experimental procedure for chronic administration involved daily oral administration (p.o.) of AG (61.65 mg/kg or 205.50 mg/kg) and caffeine (25 mg/kg) to mice (n = 5 per group) at 09:00 h. EEG and EMG recordings (using Power Lab C) were conducted at baseline (BL) on hours 0, 1, 3, and 5 after dosing as well as during the withdrawal period, following the detailed procedure described previously [10]. After two hours of recording, the mice were killed under deep anesthesia to end the study. Whole brains were removed, frozen on dry ice, and stored at −80 °C for subsequent Western blot and ELISA experiments.

### 4.3. Laboratory Analysis

Serotonin and dopamine levels in the brain were evaluated utilizing specialized mice-specific kits (BT-LABS, Shanghai, China). Measurements were conducted using a microplate spectrophotometer (Elx-800, Bio-Tek Instruments Inc., Winooski, VT, USA) adjusted to 450 ± 10 nm. The intra-assay and inter-assay CV were <8.0% and <10.0% for serotonin and dopamine, respectively. The serotonin kit had a 0.02–6 ng/mL range and 0.012 ng/mL sensitivity, and the dopamine kit had a 0.5–200 ng/mL range and 0.23 ng/mL sensitivity.

Brain samples were homogenized and sonicated in a cold Tris-HCl buffer (10 mM, pH 7.4) containing a protease and phosphatase inhibitor cocktail for Western blot analysis. The total protein content was determined using a microvolume spectrophotometer (NanoDrop, Maestrogen Inc., Taiwan). The homogenates were mixed with 2xLaemmli sample buffer and heated for five minutes. Each protein sample was separated by 12% sodium dodecyl sulfate (SDS)-polyacrylamide gel electrophoresis (PAGE) and subsequently transferred onto nitrocellulose membranes using the Power Blotter (Thermo Fisher, Waltham, MA, USA). Membranes were blocked for 1 h at room temperature in a 5% solution of bovine serum albumin in Tris-buffered saline containing 0.1% Tween to prevent non-specific binding. After the membranes were incubated at 4 °C with primary antibodies targeting GABA_A_R2, GABA_B_R1, and GABA_B_R2, EEG and EMG recordings (using Power Lab C, ADInstruments, Inc., Colorado Springs, CO, USA) were conducted at baseline on hours 0, 1, 3, and 5 after dosing as well as during the withdrawal period following the detailed procedure described previously: GluA1, GluN1, GluN2A, serotonin 1A receptor (5-HT1A), brain-derived neurotrophic factor (BDNF), glial fibrillary acidic protein (GFAB), and nerve growth factor (NGF) (Abcam, Cambridge, UK). The membranes underwent treatment with an HRP-linked secondary antibody for two hours at room temperature the following day. The β-actin antibody (Sigma, St. Louis, MO, USA) standardized protein loading. The diaminobenzidine substrate technique was employed to visualize antibody interactions. Subsequently, band analysis was conducted using Image J software, 1.49, Java Program, National Institutes of Health, Bethesda, MD, USA) for densitometric evaluation.

### 4.4. Statistical Analyses

The statistical package SPSS (version 22.0, IBM, Armonk, NY, USA) was used to evaluate the data. The Shapiro–Wilk and Levene’s tests were used to examine the data’s normality and the variances’ homogeneity. A one-way analysis of variance (ANOVA) was performed to evaluate the differences between groups, followed by Tukey’s post hoc test. Data were presented as mean ± standard deviation (SD). *p*-values less than 0.05 were accepted as significant.

## Data Availability

Data are contained within the article and Supplementary Materials.

## Supplementary Materials

The following supporting information can be downloaded at https://www.mdpi.com/article/10.3390/ph17121649/s1, Figure S1: Full immunoblots related to Figure 4 on the brain tissue of mice: GABA_A_R2 (A), GABA_B_R1 (B), GABA_B_R2 (C), and β-actin (D). Each immunoblot is representative of three independent experiments. Red dotted rectangles delineate results shown in Figure 4. MW (in kDa) is indicated. Figure S2: Full immunoblots related to Figure 5 on the brain tissue of mice; GluA1 (A), GluN1 (B), GLuN2 (C), 5-HT1A (D), and β-actin (E). Each immunoblot is representative of three independent experiments. Red dotted rectangles delineate results shown in Figure 5. MW (in kDa) are indicated. Figure S3: Full immunoblots related to Figure 6 on the brain tissue of mice: GFAP (A), BDNF (B), NGF (C), and β-actin (D). Each immunoblot is representative of three independent experiments. Red dotted rectangles delineate results shown in Figure 6. MW (in kDa) are indicated.
