# Supplementary material for: The Effect of Water-Soluble Alpinia Galanga Extract on Sleep and the Activation of the GABAAergic/Serotonergic Pathway in Mice
Source: Pharmaceuticals (Basel). 2024 Dec 8;17(12):1649. doi: 10.3390/ph17121649 (PMC11728628; doi:10.3390/ph17121649)
Supplement: Supplementary file 1 [file pharmaceuticals-17-01649-s001.zip › pharmaceuticals-3296139-supplementary.pdf]

The Effect of Water-soluble Alpinia Galanga Extract on Sleep and Activation of GABAAergic/Serotonergic Pathway in Mice

Kazim Sahin [1*], Ahmet Kayhan Korkusuz[2], Emre Sahin[3] Cemal Orhan[1], Besir Er [4], Abhijeet Morde [5], Muralidhara Padigaru [5] and Ertugrul Kilic [6]

[1] Department of Animal Nutrition, Faculty of Veterinary Medicine, Firat University, 23119, Elazig, Turkiye; ksahin@firat.edu.tr (K.S.); corhan@firat.edu.tr (C.O.)
[2] Department of Physiology, School of Medicine, Istanbul Medipol University, 34810, Istanbul, Turkiye; akayhankorkusuz@gmail.com (A.K.K.); ertugaldatmaz@hotmail.com (İ.E.A.)
[3] Department of Animal Nutrition, Faculty of Veterinary Medicine, Bingol University, 1200, Bingol, Turkiye; esahin@bingol.edu.tr (E.S.);
[4] Department of Biology, Faculty of Science, Firat University, 23119, Elazig, Turkiye; hgencoglu@firat.edu.tr (H.G.); ber@firat.edu.tr (B.E.)
[5] Research and Development, OmniActive Health Technologies, Mumbai, India; a.morde@omniactives.com (A.A.M); m.padigaru@omniactives.com (M.P.)
[6] Department of Physiology, Faculty of Medicine, Istanbul Medeniyet University, 34700, Istanbul, Turkiye; kilic44@yahoo.com (E.K.)

* Correspondence: nsahinkm@yahoo.com; Tel.: (+90 424 237 00 00 ext: 3938)

Supplementary Figures

A) $GABA_AR2$

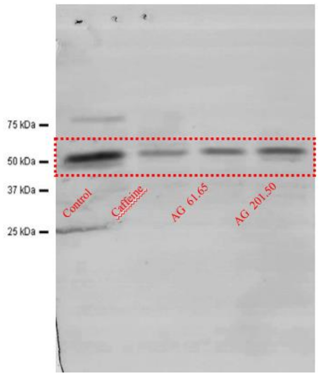

B) $GABA_BR1$

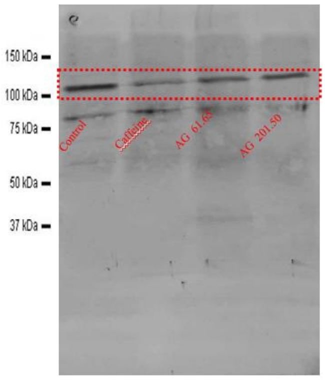

C) $GABA_BR2$

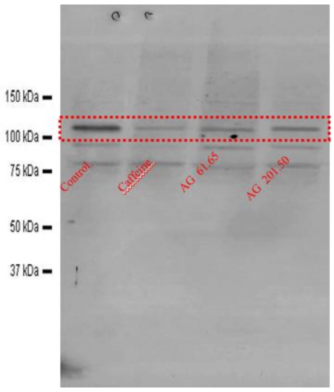

D) β-actin

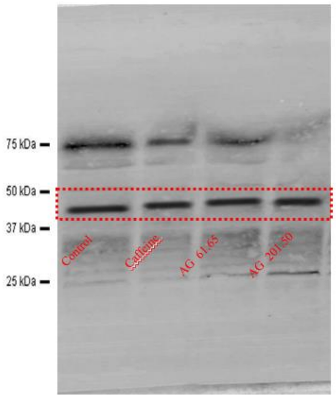

Fig. S1. Full immunoblots related to Fig. 4 on brain tissue of mice; $GABA_AR2$ (A), $GABA_BR1$ (B), $GABA_BR2$ (C), and β-actin (D). Each immunoblot is a representative of three independent experiments. Results shown in Fig.4 are delineated by red dotted rectangles. MW (in kDa) are indicated.

A) GluA1

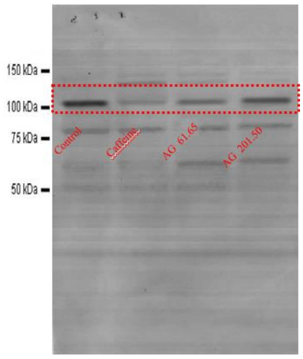

B) GluN1

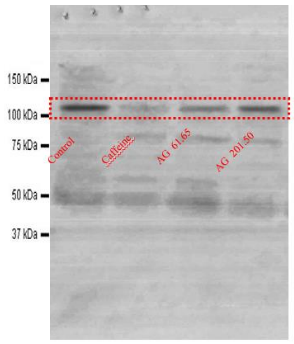

C) GLuN2

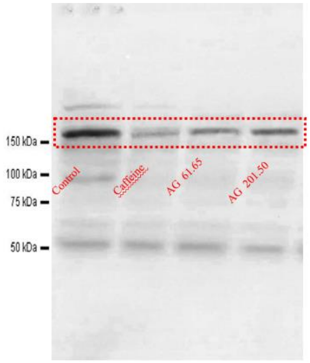

D) 5-HT1A

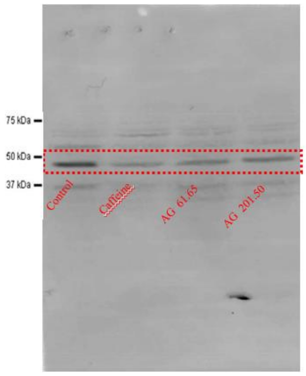

E) β-actin

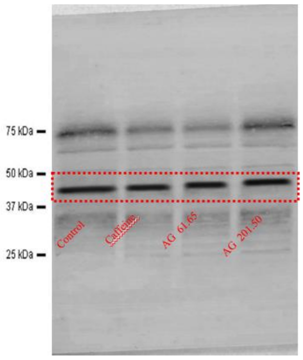

Fig. S2. Full immunoblots related to Fig. 5 on the brain tissue of mice; GluA1 (A), GluN1 (B), GLuN2 (C), 5-HT1A (D), and β-actin (E) Each immunoblot is a representative of three independent experiments. Results shown in Fig. 5 are delineated by red dotted rectangles. MW (in kDa) are indicated.

A) GFAP

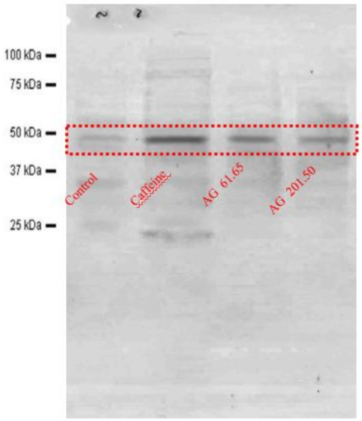

B) BDNF

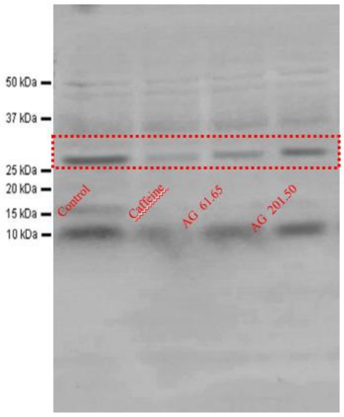

C) NGF

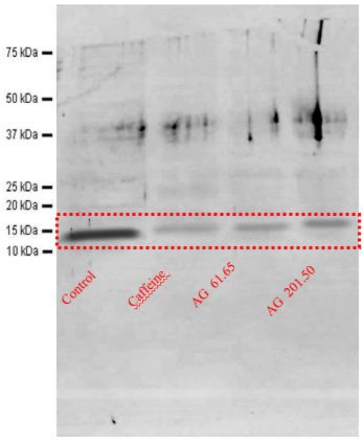

D) β-actin

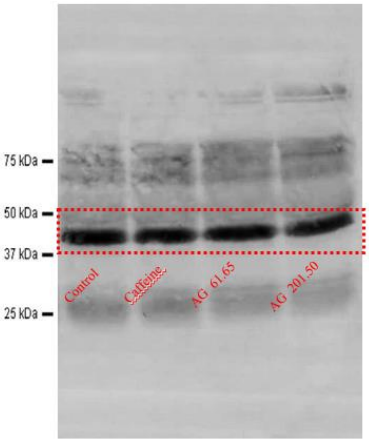

Fig. S3. Full immunoblots related to Fig. 6 on brain tissue of mice; GFAP (A), BDNF (B), NGF (C), and β-actin (D). Each immunoblot is a representative of three independent experiments. Results shown in Fig. 6 are delineated by red dotted rectangles. MW (in kDa) are indicated.
